# The benefit of the diffusion kurtosis imaging in presurgical evaluation in patients with focal MR-negative epilepsy

**DOI:** 10.1038/s41598-021-92804-w

**Published:** 2021-07-09

**Authors:** Michaela Bartoňová, Marek Bartoň, Pavel Říha, Lubomír Vojtíšek, Milan Brázdil, Ivan Rektor

**Affiliations:** 1 Central European Institute of Technology (CEITEC), Multimodal and Functional Neuroimaging Research Group, Masaryk University, Kamenice 753/5, 625 00 Brno, Czech Republic; 2 Brno Epilepsy Center, Full member of the European Reference Network (ERN) EpiCARE, First Department of Neurology, St. Anne′s University Hospital, Faculty of Medicine, Masaryk University, Brno, Czech Republic

**Keywords:** Epilepsy, Image processing

## Abstract

The effectivity of diffusion-weighted MRI methods in detecting the epileptogenic zone (EZ) was tested. Patients with refractory epilepsy (N=25) who subsequently underwent resective surgery were recruited. First, the extent of white matter (WM) asymmetry from mean kurtosis (MK) was calculated in order to detect the lobe with the strongest impairment. Second, a newly developed metric was used, reflecting a selection of brain areas with concurrently increased mean Diffusivity, reduced fractional Anisotropy, and reduced mean Kurtosis (iDrArK). A two-step EZ detection was performed as (1) lobe-specific detection, (2) iDrArK voxel-wise detection (with a possible lobe-specific restriction if the result of the first step was significant in a given subject). The method results were compared with the surgery resection zones. From the whole cohort (N=25), the numbers of patients with significant results were: 10 patients in lobe detection and 9 patients in EZ detection. From these subsets of patients with significant results, the impaired lobe was successfully detected with 100% accuracy; the EZ was successfully detected with 89% accuracy. The detection of the EZ using iDrArK was substantially more successful when compared with solo diffusional parameters (or their pairwise combinations). For a subgroup with significant results from step one (N=10), iDrArK without lobe restriction achieved 37.5% accuracy; lobe-restricted iDrArK achieved 100% accuracy. The study shows the plausibility of MK for detecting widespread WM changes and the benefit of combining different diffusional voxel-wise parameters.

## Introduction

About 70% of pharmacologically treated epileptic patients achieve seizure freedom and significant increase in quality of life. Surgical intervention became a treatment of choice for the remaining, medically refractory patients^1^. The primary aim of the surgery is to remove the area of the brain responsible for seizure genesis. Therefore, the surgery outcome relies on its precise localization. Brain imaging, especially for structural abnormalities visible on magnetic resonance imaging (MRI), has a crucial role in the presurgical assessment of patients with epilepsy. However, in 20% to 40% of surgery candidates, conventional clinical MRI examination reveals no visible or inconclusive abnormalities; such patients are considered MR-negative^2^. Assessment of this covert lesion′s location is usually done using modalities with radiation exposure, such as single-photon emission computed tomography (SPECT) or positron emission tomography (PET). Advanced neuroimaging techniques based on MRI, but beyond routine clinical protocol, such as DWI, can provide a considerable benefit in detecting previously nonvisible structural irregularities^3^, partially reducing the need to use radiation.

Diffusion-weighted imaging (DWI), a subset of MRI methods, has the unique ability to capture water molecule displacement determined by the microstructural environment, and thus to serve as a microstructure probe^4^. In patients with normal-appearing MRI, refractory epilepsy is in 42% caused by focal cortical dysplasia (FCD); a less frequent cause is hippocampal sclerosis (HS)^5^. These (FCD and HS) pathophysiological changes span a large spectrum of cortical development malformations in tissue microstructure (such as disrupted cortical lamination, cytologic and cytoarchitecture abnormalities, presence of balloon cells, and myelination changes)^6–8^ altering diffusion parameters derived from various diffusion models. Recent studies showed that multicompartment models such as NODDI^9^ and CHARMED^10^ bring significant clinical and pathophysiological benefits to study of epilepsy^11^. However, studies confirming an association with post-surgical histology are needed^12^. The most widespread and well described method in the literature remains diffusion tensor imaging (DTI) and its extension, diffusion kurtosis imaging (DKI). Despite their simplicity, many studies have reported changes in parameters derived from diffusion tensor (DT) and kurtosis tensor (KT) and presented their association with underlying histopathology in patients with epilepsy.

Barrier disruption on the microstructural level and/or cell enlargement result in less constrained water diffusion, and this phenomenon could be reflected in DWI images as increased mean diffusivity (MD). Moreover, with fewer obstacles (through disappearance due to changes in myelination and cell membranes), water diffusion comes closer to a free diffusion and the diffusion isotropy increases. In DWI, this change is represented by a decline in fractional anisotropy (FA). Furthermore, the lack of barriers and microarchitecture impairments cause water diffusion distribution more similar to Gaussian, and therefore mean kurtosis (MK) parameters tend to decrease^13^.

Previous studies have demonstrated a link between diffusion parameter changes and epilepsy. Increases in the MD corresponding to the lesion and lesion-adjacent cortical areas^11,14–16^ and directly connected white matter (WM)^17,18^ were reported. Widespread declines of FA were reported, mainly in the WM tracts directly connected to the lobe with epileptogenic tissue^11,14,19^ as well as in epilepsy-related cortical areas^20^. Moreover, recent studies have revealed that reduction in MK in both gray matter (GM) and WM is a more sensitive method for detecting impairment in patients with epilepsy than conventional diffusional methods (such as MD and FA)^11,14,21^.

Although most changes in diffusion parameters are spatially localized in areas associated with seizure generation, they are not restricted to those areas. At the global level, widespread changes were detected in epileptic patients. These changes were observed predominantly within the whole lobe, or even the hemisphere ipsilateral to the lesion when compared to contralateral areas^22–25^. These observations, based on reduced FA and/or increased MD, suggest broad lobe-/hemisphere-specific pathological changes.

Taken together, DT and KT parameters showed solid sensitivity to detecting changes associated with epilepsy. Unfortunately, the DWI methods are also characterized by a lack of specificity (detected changes frequently extend beyond the epilepsy lesion location).

Two research objectives were defined. Objective 1: to reveal the most suitable DT/KT parameter for detecting large-scale, lobe-specific impairments of deep WM associated with the epileptogenic process, and subsequently to use this parameter for selecting the lobe with the highest rate of WM asymmetry as the lobe containing the areas responsible for seizure generation. Objective 2: to design and test the effectivity of advanced DWI indexes based on concurrent occurrences of significant reductions in MK and FA and increases in MD. The new index, iDrArK (increased mean Diffusivity, reduced fractional Anisotropy, reduced mean Kurtosis), is introduced. This combined metric is based on presumed underlying microstructural pathology. As described above, it would be rather unlikely to observe changes in diffusion parameters opposite to those described above (e.g., MD reduction). As a final step, iDrArK maps were restricted to a single lobe according to the results from objective 1.

The index was designed to increase the (commonly observed) low specificity of individual parameters by combining them (objective 2) and by removing the results from unimpaired lobes (objective 1). The comparison with individual parameters, and their pairs was also evaluated as a part of objective 2. The obtained results were compared with resection areas confirmed by ILAE outcome (i.e. the outcome criterion introduced by the International League Against Epilepsy) equal to 1 and/or histopathological findings. We assume that the post-processing approach introduced in this study could enhance the DWI contribution in presurgical evaluations as a part of a broader spectrum of neuroimaging methods and thus reduce the radiation exposure that patients currently experience.

## Methods

### Subjects and image acquisition

Twenty-five medically refractory focal epilepsy surgery candidates were recruited. Inclusion criteria were as follows: patient was diagnosed as MR-negative by an experienced neuroradiologist, any structural abnormalities on MRI were classified as inconclusive (lesion could not be fully confirmed nor negated), or found abnormalities were classified as suspicious but were not in alignment with other diagnostic modalities during standard clinical presurgical evaluation imaging (e.g., ictal video-electroencephalogram). MRI sequences measured for the purposes of evaluating (non)lesionality are presented in the Supporting Information in Table S1. The acquisition protocol for structural MRI evaluation was based on the recommendations by Wellmer et al.^26^, published in *Epilepsia* in 2013, and complies with the proposal by Bernasconi et al.^27^, published in *Epilepsia* in 2019. A control group of 100 age- and sex-matched healthy individuals was recruited. A summary of surgical and demographic data of both study groups is presented in Table 1.

**Table 1:** Demographic and clinical summary of study group and control subjects.

	Patients	HC
Number of subjects	25	100
Sex [M/F]	13/12	52/48
Median age [IQR]	26y [24–36 y]	30.5y [24–37.5 y]
ILAE outcome	1(14x), 2(1x), 3(3x), 4(7x)	–
Histology	6x HS, 2x gliosis, 12x FCD, 4x negative, 1x low-grade gliomas	–

*M* male, *F* female, *IQR* interquartile range (Q1–Q3), *HS* hippocampal sclerosis, *FCD* focal cortical dysplasia, *HC* healthy controls.

All subjects signed an informed consent form and the study was approved by the Research Ethics Committee of the Masaryk University and the Ethics Committee of the St. Anne's University Hospital. This study was designed in accordance with the Declaration of Helsinki.

T1-weighted images (1 mm^3^ iso voxel) and DWIs (2 mm^3^ iso voxel, 30 diffusion directions for b-values 700 s/mm^2^, 1000 s/mm^2^, 2300 s/mm^2^, and 10 repetitions for 0 s/mm^2^; additionally, 10 non-diffusion weighted volumes with opposite phase-encoding direction) were acquired on a 3T Siemens MAGNETOM Prisma. For detailed sequence information, see Supporting Information Tables S2 and S3. All patients subsequently underwent resection surgery. The surgery target was set as an intersection of results from ictal and interictal single-photon emission computed tomography (SPECT), positron emission tomography (PET), ictal and interictal video-EEG, high density scalp electroencephalography (EEG), and neuropsychology. The surgery indication was decided by an epilepsy surgery commission composed of experienced neurologists, neurosurgeons, radiologists, and psychologists. To confirm the location, patients underwent stereoelectroencephalography (SEEG) examination prior to the surgery. Positive ILAE outcome (class 1–4) and/or histopathological findings confirmed the accuracy of a resected area. The average outcome follow-up period for patient group was 33 months, minimum 6 months.

### DWI data preprocessing and estimation of DT and KT

Diffusion MRI data were preprocessed using MRtrix 3.0^28^ and FSL 6.0 (www.fsl.fmrib.ox.ac.uk) ^29^. Preprocessing and parametric map estimation was identical for all groups. Preprocessing steps were set with respect to the recommendations reported by Maximov et al. in^30^, and consisted of the following steps: (1) noise correction using Marchenko-Pastur principal component analysis^31,32^; (2) correction for Gibbs ringing artifacts^33^; (3) motion correction, eddy current, and susceptibility distortion correction using FSL tools *eddy*^34,35^ and *topup*^30^; (4) bias field correction calculated by advanced normalization tools (ANTs)^36^; (5) spatial smoothing using Gaussian kernel with the FWHM of 2.5 mm^37^. Preprocessed DWI data were manually checked to ensure that artifact correction was successful.

Parametric maps were obtained using the DESIGNER toolbox^37^. Diffusion tensor and kurtosis tensor were estimated using DKI^38^. Three main parametric maps were calculated: MD and FA from diffusion tensor and MK from diffusion kurtosis tensor.

### Processing of DT/KT parametric maps

**Figure 1:**
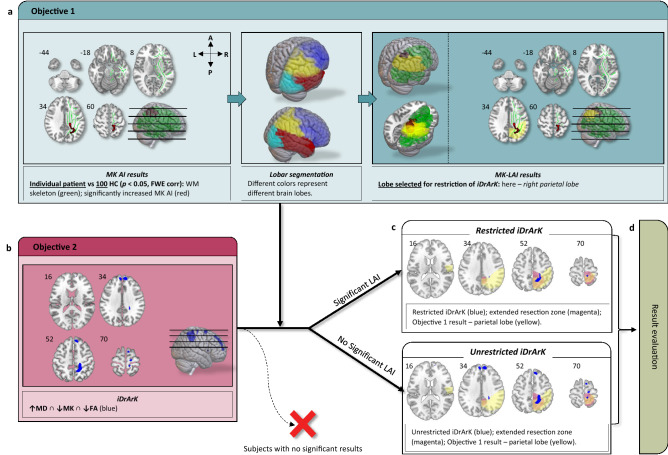
Data processing flowchart: (**a**): The Lobar Asymmetry Index (LAI) analysis for each patient was performed; (**b**): Combination iDrArK (increased mean Diffusivity, reduced fractional Anisotropy, reduced mean Kurtosis) maps were created by overlapping thresholded statistical parametric maps of increased MD, reduced FA, and reduced MK; (**c**): Lobe-specific restriction of iDrArK, if a significant result from the LAI analysis for a given patient was obtained; (**d**): Results were then compared to the resection zone extended by 10 mm in each direction. The red ″X″ represents subjects with no significant clusters in the iDrArK map (before the lobar-restriction step).

We used two-step processing of the DWI data, following the two objectives (visualized in Fig. 1).

#### Objective 1, WM asymmetry index

A symmetric WM skeleton was separately created for each subgroup one-subject-and-all-controls using the FSL tools tract-based spatial statistics (TBSS)^39^ and *tbss_sym*. In order to eliminate the partial-volume effects in superficial WM, the threshold for defining WM/GM borders was set to 0.2 during the analysis setup, resulting in a skeleton comprising rather deep WM. The MD, MK, and FA parametric maps were projected onto the symmetric skeleton and an asymmetry index (AI) was calculated for each voxel using Eq. (1):

1 $$AI_{{vx}} = \left( {right_{{vx}} - left_{{vx}} } \right)/\left( {right_{{vx}} + left_{{vx}} } \right).$$

where $vx$ stands for voxel; $right_{{vx}}$ and $left_{{vx}}$ are corresponding voxels from the right/left hemispheres. This calculation resulted in a set of values corresponding to the size of one hemisphere.

The asymmetry can be a result of increased parameter on one side or decreased on another. As mentioned in the introduction, in patients with epilepsy, MD values tend to increase, while MK and FA values decrease mainly, but not exclusively, within an impaired lobe. Therefore, increased MD asymmetry indicates impairment in the ipsilateral, right hemisphere (and conversely, decreased MD asymmetry indicates impairment in the contralateral, left hemisphere). On the other hand, increased MK/FA asymmetry indicates impairment in the contralateral, left hemisphere (and vice versa).

A two-step process of AI evaluation was implemented. First, a nonparametric permutation test using FSL's *randomise* tool was applied to compare individual patients with a control group. Sex and age were used as covariates of no interest. One-tailed two-sample *t*-tests were used to detect changes in AI derived from individual DWI parameters. The results were controlled for multiple comparisons with family-wise error (FWE) correction using threshold-free cluster enhancement, with significance level set to *P* < 0.05, and clusters with < 20 significant voxels per lobe were excluded. Secondly, the statistical results in the WM skeleton were assessed with respect to belonging to particular lobes via a Lobar Asymmetry Index (LAI) evaluation. The LAI was per each lobe calculated using Eq. (2):

2 $$LAI_{{LOBE}} = \frac{{N_{{vx}} \left( {p_{{FWE}} < 0.05} \right)_{{LOBE}} }}{{N_{{vxLOBE}} }},$$

where $N_{{vx\;LOBE}} \;{\text{and}}\;N_{{vx}} \left( {p < 0.05} \right)_{{LOBE}} ~$ represents the number of skeleton voxels within particular lobe and the number of significant voxels respectively. The lobe with the highest LAI was taken as a result of this analysis level, i.e., epilepsy-related lobe identification. The resulting lobes were compared to the location of the resection The accuracy of the whole method was assessed for each metric ($acc_{{X - LAI}}$, where *X* stands for FA, MD and MK) as number of patients with successful lobar detection (SLD) to number of patients with any significant results from impaired lobar detection (ALD) step:

3 $$acc_{{X - LAI}} = \frac{{SLD_{{X - LAI}} }}{{ALD_{{X - LAI}} ~}}.$$

Here, $~X$ stands for FA, MD and MK respectively. After the evaluation of LAIs for each DWI parameter (FA-LAI, MK-LAI, MD-LAI), the most suitable parameter was determined in terms of the ratio of patients with correctly identified lobe to all patients with significantly identified lobe. LAI results calculated using this parameter were later used to enhance EZ detection specificity in objective 2.

#### Objective 2, EZ voxel-wise, cortical detection

All estimated whole-brain voxel-wise MD, MK, and FA maps were normalized into the MNI152 standard-space through nonlinear registration in SPM12 (www.fil.ion.ucl.ac.uk). All maps were restricted using a brain mask containing cortical GM with adjacent WM and amygdala (described in detail in the Methods in the Supporting Information). The SPM12 toolbox was also used to provide statistical analysis. Voxel-wise two-sample *t*-tests comparing individual epileptic patients to a control group were performed. Sex and age were used as covariates of no interest.

**Figure 2:**
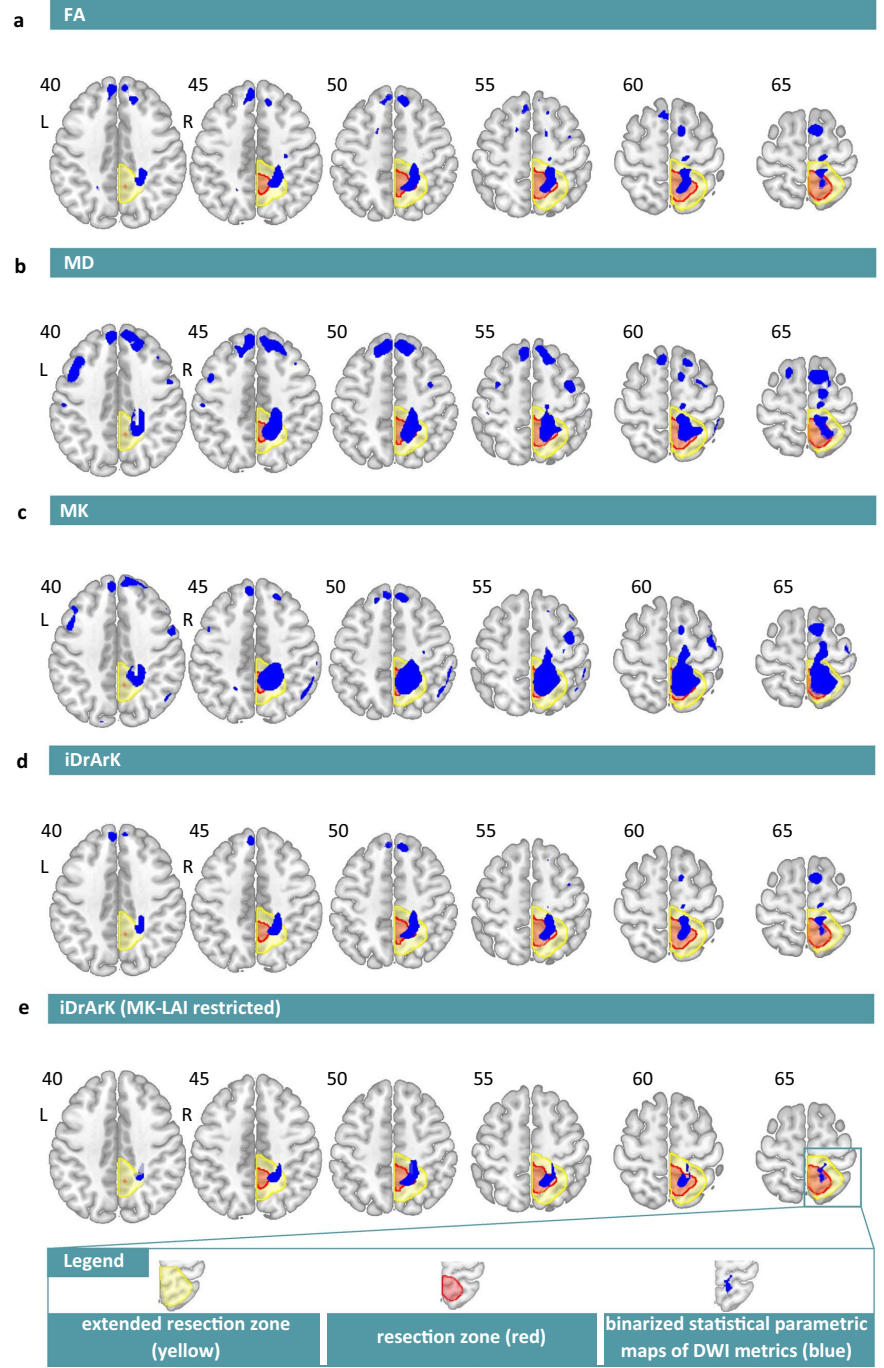
Example of binarized statistical parametric maps of DWI metrics (blue) for one subject. (**a**) FA–Fractional Anisotropy; (**b**) MD–﻿Mean Diffusivity; (**c**) MK–﻿Mean Kurtosis; (**d**) iDrArK–﻿increased mean Diffusivity, reduced fractional Anisotropy, reduced mean Kurtosis; (**e**) iDrArK with MK-LAI–﻿Lobar Asymmetry Index calculated from MK data restriction. Extended resection zone (light yellow); resection zone (red).

Binary images containing only areas with significantly increased MD, decreased FA, and decreased MK (iDrArK) for each epileptic patient were created by overlapping statistical maps (thresholded at *P* < 0.001, uncorrected for multiple comparisons). An example of metric maps and their overlap for one subject is visualized on Fig. 2.

The resulting iDrArK maps were masked by significant LAI results (if detected significantly in objective 1), i.e., maps were restricted to one brain lobe. In patients with no significant LAI results, whole-brain unrestricted iDrArK maps were used. Clusters < 20 voxels were removed. For more detailed information regarding statistical thresholding, see the Methods in the Supporting Information.

Finally, the iDrArK maps were compared with the resection zone extended by 10 mm (extended resection zone, eRZ)^40^. The extension was used to account for a brain shift that can occur after resective surgery (described in more details in the Methods in the Supporting Information). For each subject, positive predictive value (PPV) was calculated as a ratio between the number of clusters within eRZ (NC_eRZ_) and the number of all clusters (NC):

4 $$PPV_{{subj}} = \frac{{NC_{{eRZ}} }}{{NC}}.$$

EZ detection was considered successful if at least 50% of the clusters were located within the eRZ. Subjects were classified as [1] successful detection (PPV≥0.5), SD; [2] unsuccessful detection (PPV<0.5), UD; and [3] nonsignificant (no statistically significant results), NS. The accuracy of the approach $\left( {acc_{{iDrArK}} } \right)$ was calculated for whole patient group as the ratio of number of patients with successful detection to all patients with any significant results:

5 $$acc_{{iDrArK}} = ~\frac{{SD}}{{SD + UD}}.$$

Finally, the detection rate $\left( {DR_{{iDrArK}} } \right)$ was calculated as the ratio of all patients with significant results to all patients:

6 $$DR_{{iDrArK}} = \frac{{SD + UD}}{{SD + UD + NS}}.$$

PPVs and their evaluations were calculated for iDrArK as well as for single MD/MK/FA maps and for the overlaps of their pairs in order to test the potential benefit of iDrArK compared to simpler approaches (again, the increase in MD and decreases in MK and FA were used for single maps, and the overlaps). To evaluate the whole detection pipeline, lobe-restricted maps were used if this information was provided by the first step–objective 1; whole-brain maps were used if step one did not yield significant results.

#### Quantification of the utility of a two-step procedure

In designing the method described above, it was important that subjects without significant results from the first step were not unnecessarily excluded from the analysis. Therefore, in some patients, lobe-restricted data (two-step approach) were compared with the resection area; in others, whole brain data (one-step approach) were assessed. To quantify the benefit of the first step in the two-step procedure, the subset of patients with significant LAI results from objective 1 was selected. On this subset, we separately tested a one-step approach (i.e., unrestricted iDrArK maps) and a two-step approach (i.e., iDrArK maps restricted by MK-LAI results). Detection rates and accuracy were quantified, and the utility of the two-step process was evaluated.

### Ethical publication statement

We confirm that we have read the Journal's position on issues involved in ethical publication and affirm that this report is consistent with those guidelines. All subjects signed an informed consent form before entering the study. The study was approved by the Research Ethics Committee of the Masaryk University and the Ethics Committee of the St. Anne's University Hospital. This study was designed in accordance with the Declaration of Helsinki.

## Results

### Objective 1: white matter LAI

Using FA values, we identified significant changes in the WM asymmetry within the lobe corresponding ipsilaterally to resection in 5 patients. In 5 patients, the incorrect lobe or results contralateral to resection were identified. Fifteen patients had no significant lobe-specific detection.

Using MD values, we identified significant changes in the WM asymmetry within the lobe corresponding ipsilaterally to resection in 2 patients. In 2 patients, the incorrect lobe or results contralateral to resection were identified. Twenty-one patients had no lobe-specific detection.

Using MK values, we identified significant changes in the WM asymmetry within the lobe corresponding ipsilaterally to resection in 10 patients. Fifteen patients had no significant lobe-specific detection.

The MK-LAI achieved the highest accuracy when compared to FA-LAI and to MD-LAI: MK-LAI: 100%; FA-LAI: 50%; MD-LAI: 50%. Therefore, MK-LAI results were further used to enhance EZ detection specificity.

### Objective 2: EZ detection accuracy

Using restricted and unrestricted iDrArK maps, we successfully identified areas localized at eRZ in 8 patients, in 1 patient the detection was unsuccessful, and 16 patients had no significant results, resulting in 89% accuracy, and 36% detection rate. For single parameters, the following accuracy (and detection rate) was achieved: FA–﻿20.8% (96%), MD–3﻿0.4% (92%), MK–﻿20.8% (96%). The accuracy (and detection rate) for overlaps of two parameters: MD_MK–﻿46.7% (60%), MD_FA–64.3% (56%), FA_MK–35.3% (68%). All single parameters (and their dyadic combinations) exhibited worse performance than iDrArK (Table 2) in terms of accuracy despite higher detection rates.

**Table 2:** Number of subjects with *successful* (*PPV*≥0.5), *unsuccessful* (*PPV*< 0.5), or (3) *nonsignificant* (no statistically significant results) detection, when using FA/MD/MK/MD_MK/MD_FA/FA_MK/iDrArK maps restricted by MK-LAI results.

Parameter	Successful detection [No. of patients]	Unsuccessful detection [No. of patients]	Nonsignificant [No. of patients]
FA	5	19	1
MD	7	16	2
MK	5	19	1
MD_MK	7	8	10
MD_FA	9	5	11
FA_MK	6	11	8
iDrArK	**8**	**1**	**16**

*FA* fractional anisotropy, *MD* mean diffusivity, *MK* mean kurtosis, *iDrArK* increased mean Diffusivity, reduced fractional Anisotropy, reduced mean Kurtosis, *MK-LAI* Lobar asymmetry index calculated from mean kurtosis values.

The row in BOLDs denotes iDrArK - the index with the highest EZ detection accuracy.

Results for both groups are summarized in Table 2. For more details, see Tables S4, S5, S6, S7, S8, S9, and S10.

### Quantification of the utility of a two-step procedure

On a subgroup of 10 patients with lobes successfully detected by MK-LAI, the results only from the second step (i.e., unrestricted iDrArK) were as follows: 3 patients with correctly detected resection zone, 5 patients with incorrectly detected RZ, and 2 subjects with no significant results. These results reflect 37.5% accuracy and 80% detection rate. By contrast, when using a two-step approach (i.e., iDrArK maps were restricted to one lobe), 5 patients had correctly identified RZ; in 5 patients, there were no significant results (100% accuracy and 50% detection rate).

## Discussion

Improving the detection of brain areas responsible for epileptic seizure generation in patients that do not respond to pharmacotherapy and lack macroscopic structural abnormalities at the same time is a challenging topic that many researchers are trying to resolve. Due to an advancement in computational possibilities, a growing number of studies fuse multimodal data in order to achieve more accurate results through complex analytical approaches^41,42^. The detection rate of such advanced computational algorithms of course strongly depends on the accuracy of each method that enters the multimodal fusion.

In this study, we focused on a single piece of the larger puzzle, DT and KT parametric map evaluation, to investigate whether we could enhance the detection rate of these DWI parameters by combining them in the process of detecting the epileptogenic zone. According to the literature, changes detected by single DT and KT parameters tend to be located within, but not restricted to, the lesion and nearby areas^14^. This study is based on the assumption that by focusing on areas with specific changes in diffusion parameters (concurrently ↓ MK, ↓ FA, and ↑ MD), we could achieve more specific localization than by using separate diffusion parameters. Our hypothesis was tested on medically refractory epilepsy surgery candidates who underwent standard clinical presurgical MRI measurement with no visible structural abnormalities or non-conclusive results, based on an evaluation by an experienced neuroradiologist and epilepsy surgery commission members.

Two objectives were defined, differing in abnormality detection scale. Objective 1 was focused on revealing the brain lobe with areas responsible for seizure generation; objective 2 aimed to detect these areas on a subtler, voxel-wise, level. This resulted in a two-step detection process of the epileptogenic zone.

As a first step, the Lobar Asymmetry Index was calculated for each subject, using FA, MD, and MK values projected onto the WM skeleton to test whether there were differences in the detection performance of large-scale deep WM epilepsy-related alternations among different DWI metrics. MK-LAI achieved the highest accuracy, i.e., mean kurtosis showed to be the most suitable diffusion parameter to detect extensive impairment of deep WM linked to seizure generation. We observed that MK-LAI successfully identified the lobe in which resection was later performed as an impaired lobe in 10 patients of 25; no patients had incorrect identification of lobe; and 15 patients did not reach statistical significance. MK-LAI did not produce any false positive results, i.e., incorrect detection of the impaired lobe. The usage of other parameters in LAI, i.e., both MD-LAI and FA-LAI, had lower accuracy and lower rates of statistically significant results. These results are in line with previously reported findings that the MK is a more appropriate diffusional parameter than FA or MD for assessing the changes in patients with epilepsy^14,43^. The lower the MK value, the less complex the tissue microstructure is, i.e. a decrease of MK reflects a pathological disruption of cell conformity and hence a loose tissue structure. The observed changes in decreases of MK could be a reflection of neuronal degeneration or a glial cell loss, alterations known to be associated with epilepsy pathologies^44^. To the best of our knowledge, this is the first study focusing on MK in deep WM in patients with medically refractory epilepsy with negative or inconclusive results from MRI. The MK-LAI results were later used to restrict iDrArK maps. For individual patients who did not reach significance in LAI analysis, unrestricted iDrArK maps were used.

As a second step, we calculated a new diffusion metric, iDrArK, in order to reveal cortical epilepsy-related pathological foci. The iDrArK metric reveals areas with concurrent increase of MD and decreases of FA and MK. This overlapping enhances specificity by reducing the number of clusters detected by single DTI or DKI metrics. The combination of MD, MK, and FA was more specific for EZ detection than these metrics separately. By strictly focusing on areas meeting all three conditions, we increased specificity at the cost of a higher number of subjects with no significant results (i.e., decreased sensitivity). However, we consider it more reasonable to identify the potential EZ in a smaller number of subjects in favor of an increased specificity. It is necessary to consider the context of presurgical imaging, i.e., DWI is usually one of several modalities involved in the presurgical evaluation, alongside structural and functional MRI, PET, SPECT, and EEG-based methods, including specific features of high-density EEG, and intracranial video EEG, ictal/interictal measurements^45^. Therefore, a lack of significant results from one modality does not exclude a patient as a surgical candidate. A 50% cut-off for iDrArK result thresholding in final evaluation was set, considering widespread structural changes, given by a network character of focal epilepsy^46^, usually located beyond the borders of areas involved in seizure generation. Compared to the lower accuracy rate of single parameters or their pair-wise combinations, iDrArK EZ detection achieved an 89% accuracy rate using the previously mentioned 50% threshold. We conclude that the iDrArK index, based on overlapping diffusional parameters biologically relevant for epilepsy, offers an indisputable advantage.

Concerning the utility of lobe-restriction of the iDrArK, the advantage of this two-step process is apparent when compared with unrestricted results in the subset of patients in which this comparison is possible. In our data set, this was a subset of 10 patients whose results are highlighted in the Supporting Information in Tables S10 and S11 (in yellow). The one-step analysis (i.e., unrestricted iDrArK) resulted in correctly detected resection zones in 3 patients and incorrectly detected zones in 5 patients; 2 patients did not achieve significance (37.5% accuracy and 80% detection rate). By contrast, when using the two-step approach (i.e., iDrArK maps were restricted to one lobe), in 5 patients the RZ was successfully detected, and 5 patients had no significant results (100% accuracy and 50% detection rate). Based on our results, we conclude that when skipping the restriction of iDrArK maps to one lobe (according to the results from objective 1), only false positive results appear in addition (or, in the best possible scenario, the results might stay unchanged). This increases the accuracy in subjects with mixed results–results in impaired and other lobe(s) at the same time. In subjects who would have completely incorrect results located only outside the impaired lobe, such incorrect results are eliminated, and the detection procedure produces no significant detection result instead of incorrect results. Overall, we observed a considerable benefit of using both steps in detecting EZ when compared to a simple iDrArK analysis; this result further emphasizes the advantage of mean kurtosis in detecting deep WM changes associated with epilepsy.

Due to the low size of our sample (as the prevalence of MR-inconclusive epilepsy in epilepsy surgery datasets is rather low) and its heterogeneity (which naturally arose from the fact that the study focused on patients with unclear lesion location), the study does not answer more specific questions related to e.g. relationships between the spatial localization of the resection area and the detection rate of iDrArK, the distinction between epilepsy of different etiologies and their classifications etc. For the same reason, no correlation between the epilepsy duration or age at disease onset with LAI accuracy/detection rate or accuracy/detection rate of EZ detection were investigated.

Despite these limitations, we may point out (without statistical evaluation) that the only patient with incorrect detection was diagnosed with temporal lobe epilepsy (TLE), and resected in the anteromesial temporal area in the left hemisphere. The TLE, especially when associated with mesial temporal sclerosis^47^, is associated with widespread extratemporal changes in brain diffusion parameters^18,19^. We assumed that our approach could be less suitable for some cases of TLE or for patients with multi-focal epilepsy, thus the introduced EZ detection methodology should be used with this caution, and further testing in more systematically characterized patient groups is recommended.

To the best of our knowledge, this is the first study utilizing DWI metric overlap based on previously reported findings to detect EZ in epileptic patients with unclear lesion-location hypothesis from clinical MRI. The results show substantial potential for improvement of EZ detection exploiting DWI methods in MR-negative epilepsy defined via standard routine clinical MRI examination. Despite the fact that DWI scanning for DTI/DKI, and subsequent necessary computations are more demanding that common structural MRI protocol, it is noninvasive, in terms of no radiation dose exposure, when compared to PET/SPECT methods. Moreover, such scanning is not time consuming for the patient since necessary scanning sequences may be acquired in less than 15 min. The DWI approach proposed here may bring further benefits when combined with other advanced structural and functional imaging methods; nevertheless, examination of these potential multimodal fusions is beyond the scope of our study. Another perspective could also provide a comparison with novel DWI methods such as NODDI or CHARMED, or the use of the metrics derived from these models, to improve the calculation of the proposed iDrArK index. Assessment of these extended methods should be an aim of the future studies in the field.

Taken together, we showed the potential benefit of the advanced DWI index consisting of FA, MD, and MK combination. However, the accuracy of the metric and relationship between epilepsy type and MRI detection rate should be a matter of further research including different and potentially larger samples of patients.

## Supplementary Information

Supplementary Information 1.

## Data Availability

The data that support the findings of this study are available from the corresponding author upon reasonable request.
