# Supplementary material for: The benefit of the diffusion kurtosis imaging in presurgical evaluation in patients with focal MR-negative epilepsy
Source: Sci Rep. 2021 Jul 9;11:14208. doi: 10.1038/s41598-021-92804-w (PMC8270902; doi:10.1038/s41598-021-92804-w)
Supplement: Supplementary file 1 — Supplementary Information 1. [file 41598_2021_92804_MOESM1_ESM.docx]

**Supporting Information**

**Table S1:** List of MRI sequences measured for evaluation of (non)lesionality of patients.

| **MRI sequence** | **No of slices** | **TR [ms]** | **TE [ms]** | **Voxel size [mm^3^]** | **Flip angle [deg]** | **Notes** |
| --- | --- | --- | --- | --- | --- | --- |
| **T2 – weighted** (turbo spin-echo) | Transversal | 6100 | 105 | 0.49x0.49x3 | 150 |  |
| **T2* – weighted** (gradient-echo) | Transversal | 852 | 19.9 | 0.69x0.69x3.9 | 20 | transversal slices used to depict hemorrhage, calcification, and iron deposition |
| **T1 – weighted** (inversion recovery) | Transversal | 2500 | 11 | 0.69x0.69x3.3 | 150 |  |
| **T2 – weighted** (turbo inversion recovery magnitude) | Transversal | 5390 | 81 | 0.69x0.69x3.6 | 150 | Long T1 components suppressed |
| **T1 – weighted** (inversion recovery) | Coronal | 2900 | 10 | 0.5x0.5x2.2 | 150 |  |
| **T2 – weighted** (turbo inversion recovery magnitude) | Coronal | 9000 | 81 | 0.69x0.69x3.6 | 150 |  |
| **T2 – weighted** (fast spin-echo) | Para-coronal | 8000 | 52 | 0.39x0.39x2 | 150 | Slices perpendicular to hippocampus |
| **T1 – weighted** (inversion recovery prepared 3D gradient-echo) | Sagittal | 2300 | 2.33 | 1x1x1 | 8 | TI = 900 ms |
| **T1 – weighted** (inversion recovery prepared 3D dual gradient-echo) | Sagittal | 5000 | 2.97 | 1x1x1 | (Dual flip angle)  1^st^: 4; 2^nd^:5 | TI_1 = 766 ms  TI_2 = 2500 ms |
| **T2 – weighted**  (inversion recovery prepared 3D fast (turbo) spin-echo) | Sagittal | 6000 | 387 | 1x1x1 | – | TI = 1900 ms |

TR, repetition time; TE, echo time; TI, inversion time; ms, milliseconds; mm, millimeters; deg, degrees

Table S2: DWI sequence details

| MRI machine | Siemens 3T MAGNETOM Prisma |
| --- | --- |
| Sequence | Spin-echo echo-planar imaging sequence |
| TR | 9000 ms |
| TE | 78 ms |
| FoV | 224 x 224 mm^2^ |
| PAT factor | 2 |
| Mode | Multi-directional diffusion weighting (MDDW) |
| Voxel size | 2.0 x 2.0 x 2.0 mm^3^ |
| b_1_ (PE direction AP) | 700 s/mm^2^ (30 directions) |
| b_2_ (PE direction AP) | 1000 s/mm^2^ (30 directions) |
| b_3_ (PE direction AP) | 2300 s/mm^2^ (30 directions) |
| b_0_ (PE direction AP) | 0 s/mm^2^ (10 repetitions) |
| b_0_ (PE direction PA) | 0 s/mm^2^ (10 repetitions) |

TR, repetition time; TE, echo time; FoV, field of view; PE, phase encoding; AP, anterior-posterior; PA, posterior-anterior

Table S3: T1 weighted sequence details

| MRI machine | Siemens 3T MAGNETOM Prisma |
| --- | --- |
| TR | 2300 ms |
| TE | 2.34 ms |
| TI | 900 ms |
| FoV | 256 x 260 mm^2^ |
| Matrix size | 256 x 256 |
| Slice | 240 sagittal slices, slice thickness = 1 mm |
| PAT factor | 2 |
| Voxel size | 1.0 x 1.0 x 1.0 mm^3^ |

TR, repetition time; TE, echo time; TI, inversion time; FoV, field of view)

**Methods**

**Brain mask**

An additional analysis step was reducing the analysis results to in-brain mask limited only to areas we assume to be associated with epileptic seizure generation (Figure S1). The brain mask consisted of cortical GM with adjacent WM, and the amygdala. The outermost voxels of the cortical surface were excluded to avoid false detection caused by misregistration artifacts; the periventricular areas, ventricles, and cerebellum were removed as areas of no interest (with no possible information for direct EZ detection). Since debate remains whether subcortical areas play a role in seizure generation, we also excluded these structures and focused on areas allowing interpretation in terms of the direct focal localization of the cortical epileptogenic regions(Badawy, Lai, Vogrin, & Cook, 2013).


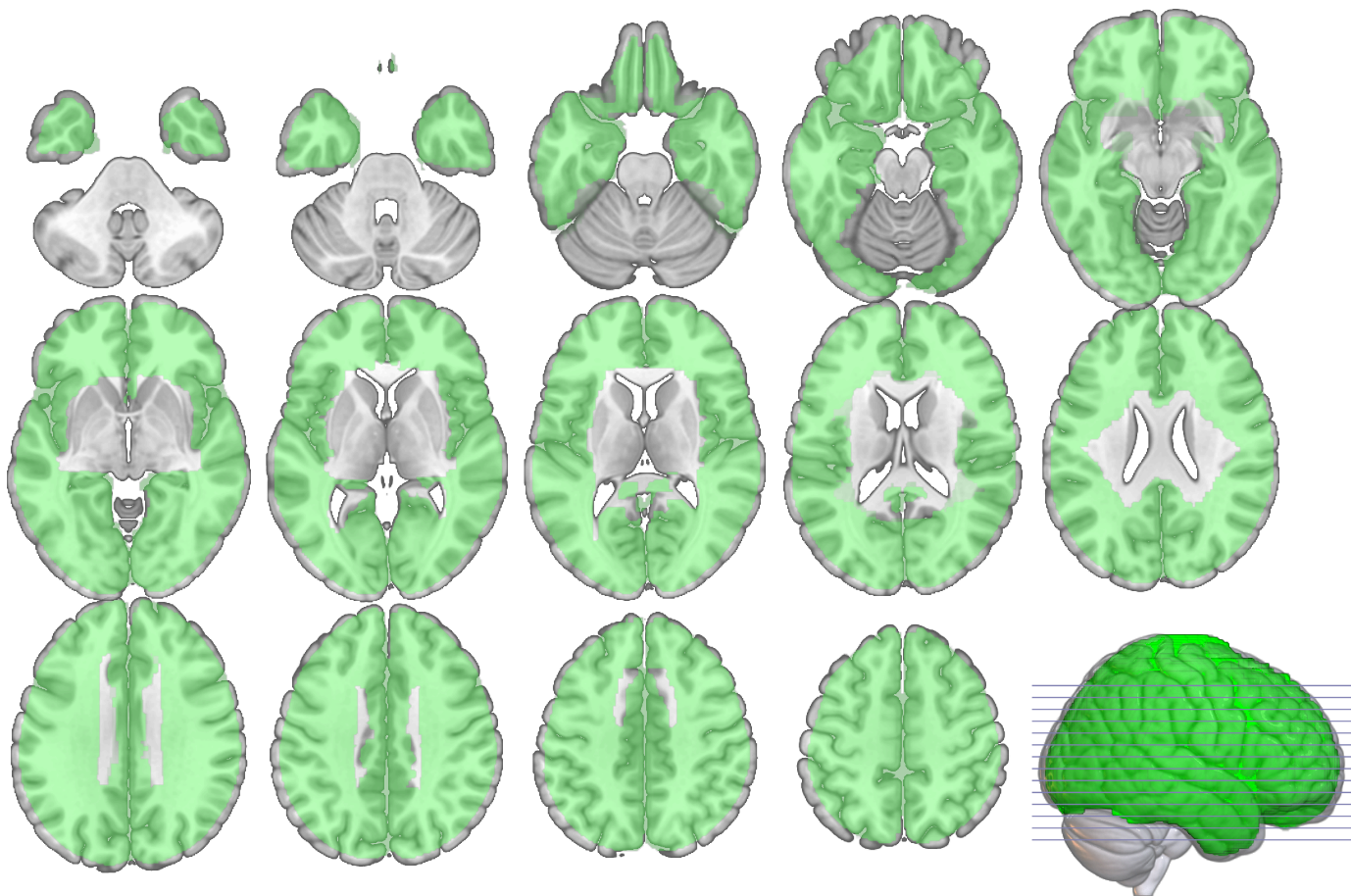


31

38

45

52

– 4

3

10

17

24

– 11

– 18

– 25

– 32

– 39

Figure S1: Mask for voxel-wise analysis in objective 2. The mask spans cortical areas (including the hippocampus) with adjacent WM, and the amygdala.

Extended resection zone (eRZ)

Initially, the extended resection zone (eRZ) was created by extending the resection zone (RZ) by 10 mm in all directions. Next, we took into account the lobe-specific location of RZ and restricted eRZ accordingly (e.g. RZ in the right frontal lobe was enlarged only within the right frontal lobe and did not cross the lateral sulcus into the temporal lobe). This resulted in an extension respecting the anatomical lobe boundaries.

**Thresholding**

We used two different thresholds for objective 1 (deep WM asymmetries computed via symmetric skeleton), and objective 2 (voxel-wise multiparametric overlaps). This dissimilarity is due to the inherently different nature of the two methods and their results. When assessing the significance in skeleton-based results, the p<0.05 with threshold-free cluster enhancement FWE correction is paradoxically less strict than p<0.001 uncorrected for multiple comparisons, and provided meaningful results; by contrast, the uncorrected thresholding led to no significant results in any subjects. On the other hand, in voxel-wise whole-brain analysis, the p<0.001 uncorrected thresholding produced more meaningful results. For such data, this threshold is quite liberal and may lead to many false positives given the high number of analyzed voxels. Nevertheless, the overlapping of the different parametric maps eliminates the extent of results that reach statistical significance in iDrArK and hence controls the false positive rate. In order to reduce spatially nonsystematic false positive results in both objectives, additional thresholding for minimum cluster size of 20 voxels was applied (considering the putative epilepsy-related pathological tissue volume).

Results

Table S4: Number of clusters in MK-LAI restricted FA maps compared to eRZ

| ID | No. of clusters in iDrArK | No. of clusters within eRZ | No. of clusters outside eRZ | Accuracy [%] |
| --- | --- | --- | --- | --- |
| 1 | 3 | 1 | 2 | 33 |
| 2 | 9 | 0 | 9 | 0 |
| 3 | 15 | 0 | 15 | 0 |
| 4 | 4 | 0 | 4 | 0 |
| 5 | 13 | 1 | 12 | 8 |
| 6 | 1 | 0 | 1 | 0 |
| 7 | 9 | 3 | 6 | 33 |
| 8 | 1 | 0 | 1 | 0 |
| 9 | 13 | 1 | 12 | 8 |
| 10 | 9 | 0 | 9 | 0 |
| 11 | 6 | 1 | 5 | 17 |
| 12 | 4 | 0 | 4 | 0 |
| 13 | 5 | 1 | 4 | 20 |
| 14 | 10 | 1 | 9 | 10 |
| 15 | 7 | 4 | 3 | 57 |
| 16 | 7 | 2 | 5 | 29 |
| 17 | 2 | 1 | 1 | 50 |
| 18 | 1 | 1 | 0 | 100 |
| 19 | 22 | 3 | 19 | 14 |
| 20 | 3 | 1 | 2 | 33 |
| 21 | 3 | 0 | 3 | 0 |
| 22 | 0 | 0 | 0 | – |
| 23 | 4 | 2 | 2 | 50 |
| 24 | 28 | 2 | 26 | 7 |
| 25 | 4 | 2 | 2 | 50 |

MK-LAI, lobar asymmetry index calculated using mean kurtosis values; eRZ, extended resection zone; FA, fractional anisotropy

Table S5: Number of clusters in MK-LAI restricted MD maps compared to eRZ

| ID | No. of clusters in iDrArK | No. of clusters within eRZ | No. of clusters outside eRZ | Accuracy [%] |
| --- | --- | --- | --- | --- |
| 1 | 2 | 0 | 2 | 0 |
| 2 | 47 | 2 | 45 | 4 |
| 3 | 3 | 0 | 3 | 0 |
| 4 | 22 | 1 | 21 | 5 |
| 5 | 4 | 1 | 3 | 25 |
| 6 | 6 | 0 | 6 | 0 |
| 7 | 9 | 2 | 7 | 22 |
| 8 | 1 | 1 | 0 | 100 |
| 9 | 38 | 5 | 33 | 13 |
| 10 | 4 | 2 | 2 | 50 |
| 11 | 0 | 0 | 0 | – |
| 12 | 2 | 0 | 2 | 0 |
| 13 | 27 | 1 | 26 | 4 |
| 14 | 16 | 1 | 15 | 6 |
| 15 | 7 | 2 | 5 | 29 |
| 16 | 6 | 1 | 5 | 17 |
| 17 | 3 | 2 | 1 | 67 |
| 18 | 1 | 1 | 0 | 100 |
| 19 | 9 | 2 | 7 | 22 |
| 20 | 0 | 0 | 0 | – |
| 21 | 36 | 0 | 36 | 0 |
| 22 | 3 | 3 | 0 | 100 |
| 23 | 2 | 1 | 1 | 50 |
| 24 | 22 | 4 | 18 | 18 |
| 25 | 2 | 2 | 0 | 100 |

MK-LAI, lobar asymmetry index calculated using mean kurtosis values; eRZ, extended resection zone; MD, mean diffusivity

Table S6: Number of clusters in MK-LAI restricted MK maps compared to eRZ

| ID | No. of clusters in iDrArK | No. of clusters within eRZ | No. of clusters outside eRZ | Accuracy [%] |
| --- | --- | --- | --- | --- |
| 1 | 16 | 1 | 15 | 6 |
| 2 | 18 | 1 | 17 | 6 |
| 3 | 4 | 0 | 4 | 0 |
| 4 | 1 | 0 | 1 | 0 |
| 5 | 33 | 1 | 32 | 3 |
| 6 | 6 | 2 | 4 | 33 |
| 7 | 5 | 2 | 3 | 40 |
| 8 | 6 | 3 | 3 | 50 |
| 9 | 10 | 3 | 7 | 30 |
| 10 | 5 | 2 | 3 | 40 |
| 11 | 0 | 0 | 0 | – |
| 12 | 1 | 0 | 1 | 0 |
| 13 | 8 | 0 | 8 | 0 |
| 14 | 7 | 0 | 7 | 0 |
| 15 | 5 | 2 | 3 | 40 |
| 16 | 3 | 1 | 2 | 33 |
| 17 | 2 | 2 | 0 | 100 |
| 18 | 4 | 1 | 3 | 25 |
| 19 | 32 | 2 | 30 | 6 |
| 20 | 1 | 1 | 0 | 100 |
| 21 | 9 | 0 | 9 | 0 |
| 22 | 1 | 1 | 0 | 100 |
| 23 | 3 | 2 | 1 | 67 |
| 24 | 42 | 4 | 38 | 10 |
| 25 | 5 | 2 | 3 | 40 |

MK-LAI, lobar asymmetry index calculated using mean kurtosis values; eRZ, extended resection zone; MK, mean kurtosis

Table S7: Number of clusters in MK-LAI restricted MD_MK maps compared to eRZ

| ID | No. of clusters in iDrArK | No. of clusters within eRZ | No. of clusters outside eRZ | Accuracy [%] |
| --- | --- | --- | --- | --- |
| 1 | 0 | 0 | 0 | – |
| 2 | 11 | 0 | 11 | 0 |
| 3 | 0 | 0 | 0 | – |
| 4 | 0 | 0 | 0 | – |
| 5 | 1 | 1 | 0 | 100 |
| 6 | 3 | 0 | 3 | 0 |
| 7 | 1 | 1 | 0 | 100 |
| 8 | 1 | 1 | 0 | 100 |
| 9 | 8 | 2 | 6 | 25 |
| 10 | 1 | 0 | 1 | 0 |
| 11 | 0 | 0 | 0 | – |
| 12 | 0 | 0 | 0 | – |
| 13 | 0 | 0 | 0 | – |
| 14 | 1 | 0 | 1 | 0 |
| 15 | 2 | 2 | 0 | 100 |
| 16 | 3 | 1 | 2 | 33 |
| 17 | 1 | 1 | 0 | 100 |
| 18 | 1 | 1 | 0 | 100 |
| 19 | 0 | 0 | 0 | – |
| 20 | 0 | 0 | 0 | – |
| 21 | 9 | 0 | 9 | 0 |
| 22 | 0 | 0 | 0 | – |
| 23 | 1 | 1 | 0 | 100 |
| 24 | 11 | 3 | 8 | 30 |
| 25 | 0 | 0 | 0 | – |

MK-LAI, lobar asymmetry index calculated using mean kurtosis values; eRZ, extended resection zone; MD_MK, maps created by overlap of areas with increased mean diffusivity and reduced mean kurtosis;

Table S8: Number of clusters in MK-LAI restricted MD_FA maps compared to eRZ

| ID | No. of clusters in iDrArK | No. of clusters within eRZ | No. of clusters outside eRZ | Accuracy [%] |
| --- | --- | --- | --- | --- |
| 1 | 0 | 0 | 0 | – |
| 2 | 5 | 0 | 5 | 0 |
| 3 | 0 | 0 | 0 | – |
| 4 | 0 | 0 | 0 | – |
| 5 | 1 | 1 | 0 | 100 |
| 6 | 0 | 0 | 0 | – |
| 7 | 1 | 1 | 0 | 100 |
| 8 | 0 | 0 | 0 | – |
| 9 | 2 | 1 | 1 | 50 |
| 10 | 0 | 0 | 0 | – |
| 11 | 0 | 0 | 0 | – |
| 12 | 1 | 0 | 1 | 0 |
| 13 | 0 | 0 | 0 | – |
| 14 | 2 | 0 | 2 | 0 |
| 15 | 2 | 2 | 0 | 100 |
| 16 | 3 | 2 | 1 | 67 |
| 17 | 2 | 2 | 0 | 100 |
| 18 | 1 | 1 | 0 | 100 |
| 19 | 1 | 0 | 1 | 0 |
| 20 | 0 | 0 | 0 | – |
| 21 | 1 | 0 | 1 | 0 |
| 22 | 0 | 0 | 0 | – |
| 23 | 1 | 1 | 0 | 100 |
| 24 | 3 | 2 | 1 | 67 |
| 25 | 0 | 0 | 0 | – |

MK-LAI, lobar asymmetry index calculated using mean kurtosis values; eRZ, extended resection zone; MD_FA, maps created by overlap of areas with increased mean diffusivity and reduced fractional anisotropy;

Table S9: Number of clusters in MK-LAI restricted FA_MK maps compared to eRZ

| ID | No. of clusters in iDrArK | No. of clusters within eRZ | No. of clusters outside eRZ | Accuracy [%] |
| --- | --- | --- | --- | --- |
| 1 | 0 | 0 | 0 | – |
| 2 | 2 | 0 | 2 | 0 |
| 3 | 2 | 0 | 2 | 0 |
| 4 | 0 | 0 | 0 | – |
| 5 | 4 | 1 | 3 | 25 |
| 6 | 0 | 0 | 0 | – |
| 7 | 3 | 2 | 1 | 67 |
| 8 | 1 | 0 | 1 | 0 |
| 9 | 2 | 1 | 1 | 50 |
| 10 | 0 | 0 | 0 | – |
| 11 | 0 | 0 | 0 | – |
| 12 | 1 | 0 | 1 | 0 |
| 13 | 0 | 0 | 0 | – |
| 14 | 1 | 0 | 1 | 0 |
| 15 | 1 | 1 | 0 | 100 |
| 16 | 7 | 2 | 5 | 29 |
| 17 | 1 | 1 | 0 | 100 |
| 18 | 1 | 1 | 0 | 100 |
| 19 | 9 | 1 | 8 | 11 |
| 20 | 1 | 1 | 0 | 100 |
| 21 | 1 | 0 | 1 | 0 |
| 22 | 0 | 0 | 0 | – |
| 23 | 3 | 1 | 2 | 33 |
| 24 | 19 | 1 | 18 | 5 |
| 25 | 0 | 0 | 0 | – |

MK-LAI, lobar asymmetry index calculated using mean kurtosis values; eRZ, extended resection zone; FA_MK, maps created by overlap of areas with reduced fractional anisotropy and reduced mean kurtosis;

**Table S10:** Number of clusters in MK-LAI restricted iDrArK maps compared to eRZ

(Yellow – subjects with significant MK-LAI results; Green – subjects that benefit from two-step approach)

| ID | No. of clusters in iDrArK | No. of clusters within eRZ | No. of clusters outside eRZ | Accuracy [%] |
| --- | --- | --- | --- | --- |
| 1 | 0 | 0 | 0 | – |
| 2 | 0 | 0 | 0 | – |
| 3 | 0 | 0 | 0 | – |
| 4 | 0 | 0 | 0 | – |
| 5 | 1 | 1 | 0 | 100 |
| 6 | 0 | 0 | 0 | – |
| 7 | 1 | 1 | 0 | 100 |
| 8 | 0 | 0 | 0 | – |
| 9 | 2 | 1 | 1 | 50 |
| 10 | 0 | 0 | 0 | – |
| 11 | 0 | 0 | 0 | – |
| 12 | 0 | 0 | 0 | – |
| 13 | 0 | 0 | 0 | – |
| 14 | 0 | 0 | 0 | – |
| 15 | 2 | 2 | 0 | 100 |
| 16 | 3 | 2 | 1 | 67 |
| 17 | 1 | 1 | 0 | 100 |
| 18 | 1 | 1 | 0 | 100 |
| 19 | 0 | 0 | 0 | – |
| 20 | 0 | 0 | 0 | – |
| 21 | 1 | 0 | 1 | 0 |
| 22 | 0 | 0 | 0 | – |
| 23 | 1 | 1 | 0 | 100 |
| 24 | 0 | 0 | 0 | – |
| 25 | 0 | 0 | 0 | – |

MK-LAI, lobar asymmetry index calculated using mean kurtosis values; eRZ, extended resection zone; iDrArK, maps created by overlap of areas with increased mean diffusivity, reduced fractional anisotropy and reduced mean kurtosis;

**Table S11:** Number of clusters in unrestricted iDrArK maps compared to eRZ.

| ID | No. of clusters in iDrArK | No. of clusters within eRZ | No. of clusters outside eRZ | Accuracy [%] |
| --- | --- | --- | --- | --- |
| 1 | 0 | 0 | 0 | – |
| 2 | 0 | 0 | 0 | – |
| 3 | 0 | 0 | 0 | – |
| 4 | 0 | 0 | 0 | – |
| 5 | 1 | 1 | 0 | 100 |
| 6 | 2 | 0 | 2 | 0 |
| 7 | 1 | 1 | 0 | 100 |
| 8 | 7 | 0 | 7 | 0 |
| 9 | 2 | 1 | 1 | 50 |
| 10 | 0 | 0 | 0 | – |
| 11 | 0 | 0 | 0 | – |
| 12 | 0 | 0 | 0 | – |
| 13 | 0 | 0 | 0 | – |
| 14 | 0 | 0 | 0 | – |
| 15 | 2 | 2 | 0 | 100 |
| 16 | 15 | 2 | 13 | 13 |
| 17 | 1 | 1 | 0 | 100 |
| 18 | 8 | 1 | 7 | 13 |
| 19 | 0 | 0 | 0 | – |
| 20 | 0 | 0 | 0 | – |
| 21 | 1 | 0 | 1 | 0 |
| 22 | 0 | 0 | 0 | – |
| 23 | 2 | 1 | 1 | 50 |
| 24 | 0 | 0 | 0 | – |
| 25 | 3 | 0 | 3 | 0 |

(Yellow – subjects with significant MK-LAI results; Green – subjects that benefit from two-step approach)

MK-LAI, lobar asymmetry index calculated using mean kurtosis values; eRZ, extended resection zone; iDrArK, maps created by overlap of areas with increased mean diffusivity, reduced fractional anisotropy and reduced mean kurtosis;

**Supplementary material reference:**

Badawy, R. A. B., Lai, A., Vogrin, S. J., & Cook, M. J. (2013). Subcortical epilepsy? *Neurology*, *80*(20), 1901‑1907. https://doi.org/10.1212/WNL.0b013e3182929f4f
